# A Unique Body Floss Technique for Reduction of Sealing Loss on the Lesser-Curvature Side in Thoracic Endovascular Repair

**DOI:** 10.1016/j.atssr.2025.08.008

**Published:** 2025-08-29

**Authors:** Koki Ikemoto, Tomoyuki Goto, Takaaki Hayashi, Masanori Yamashita, Katsuhiko Oka, Akiyuki Takahashi

**Affiliations:** 1 Department of Cardiovascular Surgery, Japanese Red Cross Society Kyoto Daiichi Hospital, Kyoto, Japan; 2 Department of Cardiovascular Surgery, Japanese Red Cross Society Kyoto Daini Hospital, Kyoto, Japan

## Abstract

It remains challenging to reduce sealing loss on the proximal lesser-curvature side in thoracic endovascular repair of blunt traumatic thoracic aortic injuries without covering the left subclavian artery. We describe a unique body floss technique for reducing sealing loss during proximal landing. In this technique, the conformable GORE TAG (W. L. Gore & Associates) can be positioned closer to the left subclavian artery and deployed more horizontally compared with the conventional procedure, which allows the device to fit better into the aortic wall. This technique is a promising strategy for treating blunt traumatic thoracic aortic injuries.

Blunt traumatic thoracic aortic injury (BTTAI) is frequently lethal in patients with trauma, and the most common sites of BTTAI are the isthmus and descending aorta.^1^ Thoracic endovascular aortic repair (TEVAR) is recommended over open surgery in patients who meet the indication for repair of BTTAI.^2^ Although the left subclavian artery (LSA) is often covered in patients with BTTAI to prevent the development of proximal endoleaks, LSA coverage causes an increase in ischemic events compared with cases with the LSA uncovered.^3^ However, assuring sufficient sealing length on the lesser-curvature side of the proximal neck without covering the LSA is often challenging. Herein, we present a unique body floss technique for reducing lesser-curvature sealing loss in TEVAR using a conformable GORE TAG (CTAG; W. L. Gore & Associates) in a patient with aortic isthmus injury.

## Technique

**Figure 1:**
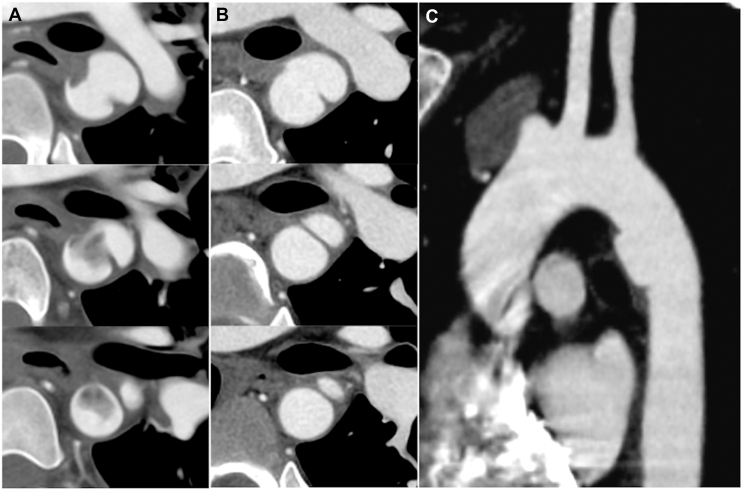
Preoperative computed tomography images. (A) An aortic injury is observed at the aortic isthmus with some blood clots within the aortic wall. (B) These disappeared at the 1-month follow-up examination. (C) The aortic injury is located on the lesser-curvature side.

A 20-year-old man who fell from a 5 m height was rushed to a hospital. Computed tomography (CT) revealed a Chance fracture of the L4 vertebra and bilateral lower leg fractures. Additionally, it revealed an aortic injury at the isthmus with blood clots (Figure 1), which was classified as grade 2 based on the classification system for BTTAIs.^2^ Careful follow-up without surgical intervention for the aortic injury was decided upon. After admission, open reduction and internal fixation and posterior spinal fusion were performed for the bilateral lower leg fractures and Chance fracture of the L4 vertebra, respectively. At the 1-month follow-up, CT revealed that the blood clots had disappeared rather than increased; hence, the patient underwent surgery for the aortic injury. Although open surgery was considered, vertebral and limb fractures could disturb the patient’s postoperative rehabilitation, and surgical invasion could cause complications around the surgery; therefore, TEVAR was selected.

**Figure 2:**
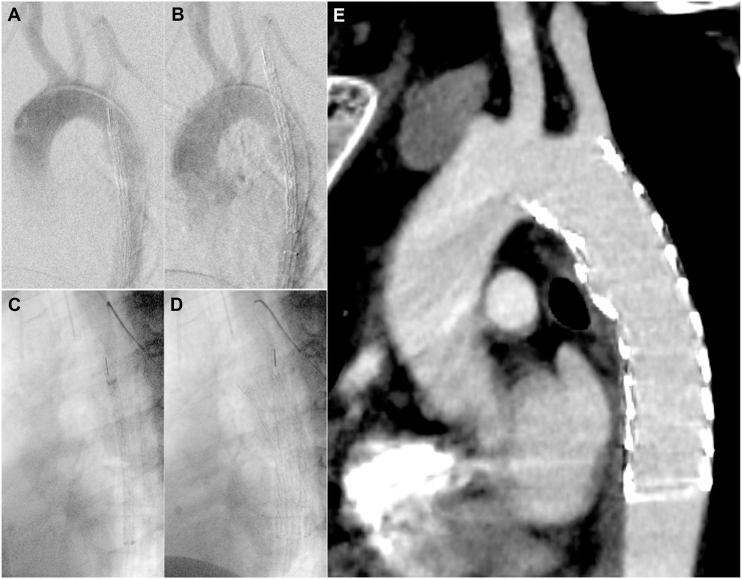
Fluoroscopic and postoperative computed tomography images. (A, B) After the proximal neck, the left subclavian artery (LSA) orifice and the site of aortic injury are confirmed using aortography, and the conformable GORE TAG (CTAG) (W. L. Gore & Associates) is carefully placed in the LSA. (C, D) The CTAG is positioned where its proximal gold marker is immediately distal to the LSA and then deployed. (E) The proximal landing length on the lesser-curvature side is adequate.

TEVAR was performed using the right common femoral artery approach. After heparinization, a radifocus guidewire (0.035-in, 400-cm, Terumo) was inserted into the right femoral artery and externalized from the left brachial artery; the body floss system was then established. After the CTAG (TGMR312610J) was delivered to the descending aorta, the orifice of the LSA, site of injury, and the proximal neck were confirmed by aortography (Figure 2A). The tip of the CTAG was gently placed in the LSA (Figure 2B). A proximal alignment marker, which is shown on the greater-curvature side in the conventional procedure, was observed on the lesser-curvature side at that time. After positioning with its proximal gold band marker immediately distal to the LSA orifice, the CTAG was carefully deployed (Figures 2C, 2D) (Supplemental Video). Finally, aortography indicated successful exclusion of BTTAI without any endoleaks. Postoperative CT showed that the proximal landing length of the device on the lesser-curvature side was adequate (Figure 2E). The patient was transferred to another hospital for rehabilitation on postoperative day 10.

## Comment

**Figure 3:**
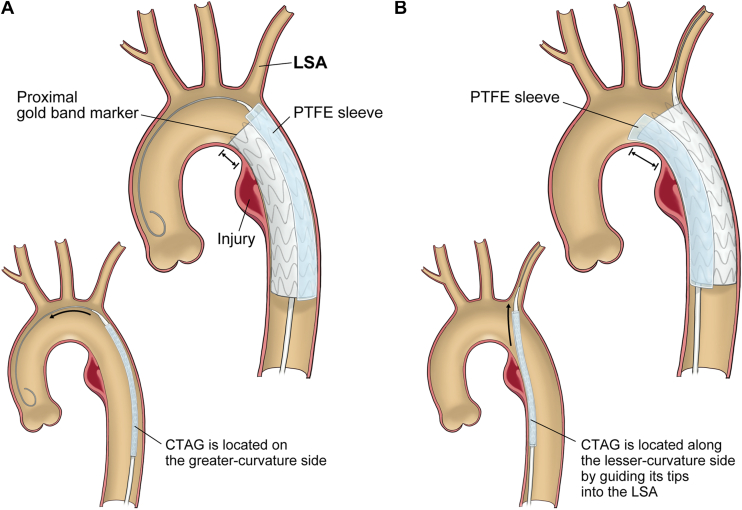
Technical images of the conventional and unique procedures. (A) In the conventional procedure, the conformable GORE TAG (CTAG) (W. L. Gore & Associates) is deployed almost perpendicular to the aorta along the wire laid on the ascending aorta, and it must be positioned slightly distal to the left subclavian artery (LSA) to prevent the polytetrafluoroethylene (PTFE) sleeve from covering the LSA. This often causes shortening of the proximal landing length on the lesser-curvature side. (B) Using our technique, it is deployed more horizontally and can be positioned closer to the LSA, so that the proximal neck fitting on the lesser-curvature side is obtained better compared with the conventional procedure.

Our unique body floss technique has some advantages in TEVAR for BTTAI, especially at the isthmus, as described in Figure 3. First, it can reduce the proximal sealing loss on the lesser-curvature side in TEVAR. In this technique, the CTAG is deployed along the lesser-curvature by guiding its tip into the LSA, making it easy to ensure a sufficient sealing length on the lesser-curvature side without covering the LSA orifice because CTAG is deployed more horizontally compared with the conventional procedure. Second, it avoids coverage of the LSA orifice with a polytetrafluoroethylene (PTFE) sleeve that belongs to the CTAG on the greater-curvature side. A case of incidental coverage of the LSA orifice by a PTFE sleeve was recently reported.^4^ Additionally, the CTAG must be located with its proximal gold band marker slightly distal from the orifice to avoid covering the LSA orifice with the PTFE sleeve, which is one of the reasons why the lesser-curvature proximal landing length is shortened. In our technique, the PTFE sleeve is naturally located on the lesser-curvature side because the device is delivered along the body floss wire, turning in the opposite direction to the aortic arch, which reduces the possibility of incidental coverage of the LSA orifice. Finally, this technique does not require any special or complicated procedures. Once the body floss system from the femoral artery to the left brachial artery is established, the CTAG is delivered automatically in the opposite direction to usual according to its properties. Therefore, the operator can focus on the proximal landing procedure.

However, this technique has 2 potential drawbacks. First, the proximal end of the CTAG on the lesser-curvature side may be at a small distance from the aortic wall, which may lead to type I endoleak. To prevent this complication, it is necessary to ensure sufficient fitting between the device and aortic wall. Hence, it is a viable option to select a larger size than usual. Second, the tip of the CTAG may injure the LSA intima. This is more likely when a body-floss wire from the aorta to the LSA describes a sharp angle. To reduce the risk of this complication, the CTAG must be handled more gently around the LSA. Furthermore, it is important to determine the indications for this technique by considering the form around the aortic arch.

In conclusion, we introduced a unique body floss technique that can reduce sealing loss on the lesser-curvature side and avoid incidental coverage of the LSA with the PTFE sleeve in TEVAR for BTTAI. This technique is a promising strategy for BTTAI of the isthmus, although its indications should be considered carefully because of potential complications.
